# Controlled Drug Delivery by Polylactide Stereocomplex Micelle for Cervical Cancer Chemotherapy

**DOI:** 10.3389/fphar.2018.00930

**Published:** 2018-08-14

**Authors:** Kai Niu, Yunming Yao, Ming Xiu, Chunjie Guo, Yuanyuan Ge, Jianmeng Wang

**Affiliations:** ^1^Department of Otorhinolaryngology Head and Neck Surgery, The First Hospital of Jilin University, Changchun, China; ^2^Department of Abdominal Ultrasound, The First Hospital of Jilin University, Changchun, China; ^3^Department of Intensive Care Unit, The First Hospital of Jilin University, Changchun, China; ^4^Department of Radiology, The First Hospital of Jilin University, Changchun, China; ^5^Department of Geriatrics, The First Hospital of Jilin University, Changchun, China

**Keywords:** controlled drug delivery, chemotherapy, cervical carcinoma, enhanced stability, stereocomplex polylactide micelle

## Abstract

A stable doxorubicin (DOX)-loaded stereocomplex micelle drug delivery system was developed via the stereocomplex interaction between enantiomeric 4-armed poly(ethylene glycol)–poly(D-lactide) and poly(ethylene glycol)–poly(L-lactide) to realize control drug release and improve tumor cell uptake for efficient cervical carcinoma therapy. All these DOX-loaded micelles including poly(D-lactide)-based micelle (PDM/DOX), poly(L-lactide)-based micelle (PLM/DOX), and stereocomplex micelle (SCM/DOX) exhibited appropriate sizes of ∼100 nm for the enhanced permeability and retention (EPR) effect. In addition, compared to PDM/DOX and PLM/DOX, SCM/DOX exhibited the slowest DOX releaser, highest tumor cell uptake and the most efficient tumor cell suppression *in vitro*. Moreover, the excellent tumor inhibiting rates of the DOX-loaded micelles, especially SCM/DOX, were verified in the U14 cervical carcinoma mouse model. Increased tumorous apoptosis and necrosis areas were observed in the DOX-loaded micelles treatment groups, especially the SCM/DOX group. In addition, all these DOX-loaded micelles obviously alleviated the systemic toxicity of DOX. As a result, SCM can be a promising drug delivery system for the future therapy of cervical carcinoma.

## Introduction

Cervical carcinoma is still one of the main causes of cancer-related deaths of female patients’ worldwide (Ferlay et al., 2015). In clinic, even though the use of neoadjuvant radio and chemotherapy have decreased the incidence and mortality rates of cervical carcinoma, lots of patients have suffered from intrinsic and acquired resistance to the therapy (Walch-Rückheim et al., 2016). In addition, the substantial severe side effect, low bioavailability, and poor delivery efficiency of the chemotherapeutic agent are still the major clinical challenges for the therapy. To solve these deficiencies, lots of nanodrug delivery systems including micelles (Tan et al., 2017; Wang et al., 2018), liposomes (de Jong et al., 2007; Chuang et al., 2017), nanogels (Ding et al., 2013; Li S. et al., 2018; Zhang et al., 2018), quantum dots (Tao et al., 2017a), nanosheet (Tao et al., 2017b; Zhu et al., 2018), modified nanoparticles (Tao et al., 2014, 2015, 2016; Ding et al., 2017), and nanospheres (Wang et al., 2016; El-Boubbou et al., 2017) have been developed to achieve the spatiotemporally controlled drug release in the tumor sites, increase the drug accumulation in the tumor cells and alleviate the systemic toxicity (Tao et al., 2013; Rosenblum et al., 2018).

Among these nanodrug delivery systems, micelles are generally the perfect choice due to the special core–shell structures, which can make them load a wide variety of drugs with a high drug loading capacity (Ma et al., 2015; Sun et al., 2017; Zhao et al., 2017; Li D. et al., 2018). In addition, due to the appropriate volumes, micelles are very suitable as nanodrug carriers to selectively accumulate at the tumor sites through the enhanced permeation and retention effect (EPR) (Wang et al., 2015c; Chen et al., 2017a,b; Khan et al., 2017; Xu et al., 2017). However, one remaining challenge for micelles-based delivery systems is the instability of them, which often leads to premature release of payloads during the circulation in the body. Chemical cross-linking of either the core or shell is one of the traditional strategies to improve the stability of micelles. However, the application of chemical cross-linkers may unfavorably affect the bioactivity of the loaded agents and the biodegradability of the delivery system (Chen et al., 2011). As an alternative, numerous non-covalent interactions including electrostatic interactions, host-guest, hydrogen bonding and stereocomplexation have been adopted as efficient strategies to improve the stability of micelles (Kim et al., 2008; Pounder et al., 2011; Shen et al., 2017).

Stereocomplexes can be considered as physical crosslinking, which are formed by the interaction between stereoregular chains of enantiomeric polymers (Feng et al., 2017). Poly(lactide) (PLA), as a biodegradable and biocompatible aliphatic polyester, has been verified to be a typical example used for stereocomplexation. PLA has a multitude of primary structures, such as isotactic poly(L-lactic acid) (PLLA) and poly(D-lactic acid) (PDLA) and syndiotactic and atactic/heterotactic PDLLA (Li et al., 2016). It has been reported that the equimolar mixture of PDLA/PLLA could form stereocomplexes with distinctive physical and chemical stability, such as improved mechanical properties, enhanced thermal resistance, and hydrolytic stability, due to the interactions between the L-lactyl and D-lactyl unit sequences (Ikada et al., 1987; Fukushima and Kimura, 2006). Concerning the advantages of PLA stereocomplexed materials in drug delivery, various PLA-based stereocomplexed formations have been developed for the transportation and delivery of different treatment agents. For instance, Ma et al. used sequential ring-opening polymerization to successfully fabricate poly(ethylene glycol)-b-poly(L-lactic acid)-bpoly(D-lactic acid) (PEG-b-PLLA-b-PDLA) stereoblock copolymers (Ma et al., 2015). The stereoblock copolymer micelles showed higher drug loading content (DLC), slower degradation, and drug release rate. In another report, Zhao and co-workers fabricated a biodegradable stereocomplex micelles (SCMs) based on amphiphilic dextran-block-polylactide (Dex-b-PLA) for efficient intracellular drug deliveries (Zhao et al., 2013). This doxorubicin (DOX) loaded SCMs exhibited high stability and sustained release profiles *in vitro*. Wang et al. previously fabricated a DOX-loaded stereocomplex micelle (SCM/DOX) via the equimolar enantiomeric 4-armed poly(ethylene glycol)–polylactide copolymers (Wang et al., 2015a). All the DOX-loaded micelles, especially the SCM/DOX displayed proper sizes for EPR, controlled DOX release, and enhanced antitumor efficacy *in vitro*. However, the *in vivo* antitumor efficacy of these DOX-loaded micelles and whether they can alleviate the systemic toxicity of DOX are not verified.

**SCHEME 1:**
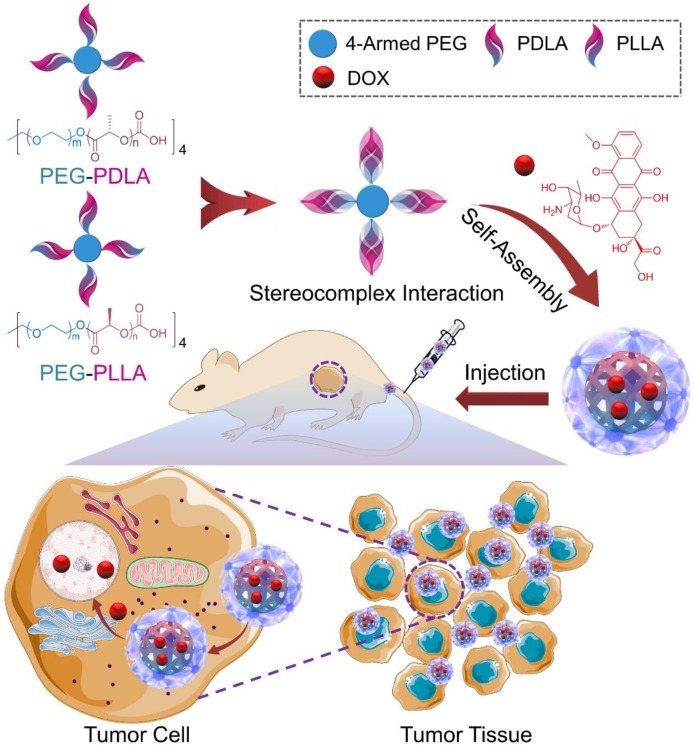
Illustration of the preparation of SCM/DOX and its passive targeting recognition (EPR effect) of the tumor cells.

In order to further confirm the *in vivo* antitumor efficacy and systemic toxicity of DOX-loaded micelles, especially the SCM/DOX toward cervical carcinoma, in the present study, an *in vivo* tumor inhibition test was evaluated on the U14 cells-bearing BALB/c mouse models (**Scheme 1**). Our results indicated that all of these DOX-loaded micelles, especially the SCM/DOX, showed satisfactory tumor suppression efficacy and a higher level of safety in comparison to free DOX⋅HCl. These DOX-loaded micelles, especially the SCM/DOX, can serve as an excellent nanoplatform for the chemotherapy of cervical carcinoma.

## Materials and Methods

### Materials

4-Armed PEG (number-average molecular weight (*M*_n_) = 10,000 Da) was purchased from Shanghai Seebio Biotech, Inc. (Shanghai, China). DLA and LLA were obtained from Changchun SinoBiomaterials Co., Ltd. (Changchun, China) and recrystallized from ethyl acetate under argon atmosphere before use. The stereocomplex micelle (SCM) was fabricated by the equimolar mixture of the enantiomeric 4-armed poly(ethylene glycol)–polylactide copolymers. Doxorubicin hydrochloride (DOX⋅HCl) was purchased from Beijing Huafeng United Technology Co., Ltd. (Beijing, China). 4^′^,6-Diamidino-2-phenylindole (DAPI), Alexa Fluor 488 phalloidin (Alexa 488), and 3-(4,5-dimethylthiazol-2-y1)-2,5-diphenyltetrazolium bromide (MTT) were purchased from Sigma-Aldrich (Shanghai, China). Human cervical cancer HeLa cells and mouse cervical cancer U14 cells were purchased from the American Type Culture Collection (ATCC). Clear 6-well and 96-well tissue culture polystyrene (TCP) plates were purchased from Corning Costar Co. (Cambridge, MA, United States). The deionized water used in this study was prepared through a Milli-Q water purification equipment (Millipore Co., MA, United States).

### Preparation of DOX-Loaded Micelles

DOX-loaded micelles were prepared through a nanoprecipitation method.(Benival and Devarajan, 2012). In briefly, DOX⋅HCl (20.9 mg) were dissolved in 6.0 mL of Milli-Q water, and then were slowly added into 10.0 mL of PEG–PLLA copolymer solution in *N*,*N*-dimethylformamide (DMF) (10.0 mg mL^-1^). After that, 2.0 mL of phosphate-buffered saline (PBS) was dropwise added into the above solution. The final solution was continuous stirring at room temperature for 12 h and subsequently dialyzed against Milli-Q water for 12 h (molecular weight cut-off (MWCO) = 3500 Da). At last, the PLM/DOX was obtained by lyophilisation. PDM/DOX and SCM/DOX were also fabricated by the same protocol.

### *In vitro* Cellular Uptake

Confocal laser scanning microscopy (CLSM) and flow cytometry (FCM) were used for quantitative analysis of cell uptake *in vitro*. CLSM. 2.0 × 10^5^ of HeLa cells were seeded on glass coverslips per well in 2.0 mL of complete high glucose Dulbecco’s modified Eagle’s medium (HG-DMEM) in 6-well plates for 24 h. PDM/DOX, PLM/DOX, SCM/DOX, or free DOX⋅HCl at a final DOX concentration of 10.0 μg mL^-1^ was added to each well. After coincubation for 2 h, the medium was removed, and the cells on glass coverslips were washed with PBS five times and fixed with 4% (W/V) PBS-buffered paraformaldehyde for 20 min at room temperature. And then, the cells were washed for five times by PBS and reacted with 0.1% (V/V) Triton X-100 in PBS for 12 min at room temperature. And then, the nuclei were then stained with DAPI for 3 min at 37 °C, after which the cells were washed with PBS five times. At last, the filamentous actin was stained with Alexa 488 for 30 min at 37°C, and washed with PBS five times. The CLSM micro-images were taken by a CLSM (LSM 780, Carl Zeiss, Jena, Germany). FCM. 2.0 × 10^5^ of HeLa cells were seeded in each well of 6-well plates and cultured for 24 h. And then, PDM/DOX, PLM/DOX, SCM/DOX, or free DOX⋅HCl at a final DOX concentration of 10.0 μg mL^-1^ was added to each well. The cells without any treatment were set as control. After a 2 h co-incubation, the medium was removed, and the cells were washed with PBS five times and then digested by trypsin. Subsequently, 1.0 mL of PBS was added and collected in centrifuge tubes. The harvested cells were centrifuged at 3000 rpm for 5 min. After removing the supernatants, the bottom cells were resuspended in 0.3 mL of PBS and examined by a flow cytometer (aaa_ex_ = 488 nm; Beckman, CA, United States).

### Cytotoxicity Assays

The cytotoxicities of DOX-loaded micelles and free DOX⋅HCl with a DOX⋅HCl concentration of 0.16–10.0 μg mL^-1^ were conducted toward HeLa cells and U14 cells by an MTT assay. In brief, 8.0 × 10^3^ cells in 180.0 μL complete HG-DMEM was planted into 96-well plates and incubated at 37°C for 24 h. And then, 20.0 μL of PBS containing various DOX formulations were added to each well and cultured for another 48 h. Subsequently, 20.0 μL of MTT at a concentration of 5.0 mg mL^-1^ was added and incubated for another 4 h. After that, the medium was carefully removed, and 150.0 μL of dimethyl sulfoxide (DMSO) was added to each well to dissolve the MTT formazan generated by the live cells. The plates were shocked for 5 min before detection. The absorbance of medium was measured at 490 nm using a Bio-Rad 680 microplate reader. The cell viability was calculated as Equation (1).

(1) $$\text{Cell viability (\%)=}\frac{\text{A sample}}{\text{A control}}\times 100$$

In Equation (1), the *A*_sample_ and *A*_control_ represented the absorbances of sample and control wells, respectively.

### *In vivo* Antitumor Efficacy Assay

Female BALB/c mice (∼4 weeks) were obtained from Vital River Laboratory Animal Center (Beijing, China). All animals were carefully treated under the guidelines approved by the Institutional Animal Care and Use Committee of Jilin University. The antitumor efficacy of DOX-loaded micelles and free DOX⋅HCl was evaluated using subcutaneous U14 cells bearing female BALB/c mouse models. Mice treated with NS (normal saline) were used as control. When the tumor volume reached about 100 mm^3^, the mice were randomly divided into 6 groups (*n* = 8 for each group) and treated with NS, free DOX⋅HCl at a DOX⋅HCl concentration of 3.0 mg [kg Body Weight (BW)]^-1^ or 6.0 mg (kg BW)^-1^, or DOX-loaded micelles at a DOX⋅HCl concentration of 3.0 mg (kg BW)^-1^ via tail vein injection every 3 days for a total of 6 doses. The groups were noted as DOX-3 and DOX-6, PDM/DOX, PLM/DOX, and SCM/DOX, respectively. The tumor sizes and body weights were monitored once-every-other-day. Tumor volume was calculated according to the following formula: tumor volume (mm^3^) = 0.5 × a × b^2^, where a and b are the largest and smallest diameter of tumor, respectively. The tumor inhibition ratio was calculated using the following formula: Tumor inhibition rate (%) = (*V*_control_-*V*_sample_)/*V*_control_ X100, where *V*_control_ and *V*_sample_ represented the tumor volumes of control and sample groups, respectively. In addition, the weights of the major organs were recorded. The organ indices of all the organs of mice were calculated according to the following formula: organ index (%) = (*w*_control_-*w*_sample_) × 100.

### *In vivo* DOX Biodistribution of Dox-Loaded Micelles at Tumor Sites

To investigate the *in vivo* DOX biodistribution of DOX-loaded micelles, female BALB/c mice bearing U14 cells (tumor volumes were about 200 mm^3^) were randomly assigned to five groups and intravenously injected with free DOX⋅HCl at a DOX⋅HCl concentration of 3.0 mg (kg BW)^-1^ or 6.0 mg (kg BW)^-1^), or DOX-loaded micelles at a DOX⋅HCl concentration of 3.0 mg (kg BW)^-1^. At 4, 12, 24, 48, and 72 h after administration, mice were sacrificed and tumors were collected, rinsed with cold PBS. Methanol was added to each tumor to extract the content of DOX. The mixture was homogenized, centrifuged, and the supernatants were collected. The amount of DOX in each tumor was determined by HPLC. The data were normalized to the tissue weight.

### Histopathological and Biochemical Analyses of Organs

The mice were sacrificed 4 days after the last treatment. After that, tumors and major organs (*i.e.*, heart, liver, spleen, lung, kidney, and sternum) were isolated, collected, were fixed in 4% (*W*/*V*) PBS-buffered paraformaldehyde overnight except sternum, and then embedded in paraffin. The organs from healthy mice were also isolated and treated as a normal control. The paraffin-embedded tumor and organ tissues were cut at a thickness of 5 μm, and prepared for hematoxylin and eosin (H&E) staining. The histopathological sections were assessed by a microscope (Nikon Eclipse *Ti*, Optical Apparatus Co., Ardmore, PA, United States). For the tumor tissues, three observation fields were evaluated to get an average value of relative necrosis area with the total area of observation field as “100%.” These data were analyzed by ImageJ software (National Institutes of Health, Bethesda, MD, United States). The relative necrotic area (%) of tumor tissues was calculated by Equation (2):

(2) $$\text{Relative necrotic area (\%)=}\frac{\text{Necrotic area in tumor section}}{\text{Total area of observed tumber section}}\times 100$$

For the histopathological assays of sternums, the isolated sternums were handled as described previously. Four paraffin sections of each sternum were performed for H&E staining.

The damages of tissues and organs were confirmed by testing the corresponding functional enzymes in blood and organs, which were detected with commercial enzyme-linked immunosorbent assay (ELISA) kits (Shanghai Lichen Biotechnology Co., Ltd., Shanghai, China). The heart indices contained creatine kinase-MB (CK-MB), creatine kinase (CK), and lactate dehydrogenase (LDH), liver-related aspartate aminotransferase (AST) and alanine aminotransferase (ALT), and kidney-associated blood urea nitrogen (BUN) and creatinine (Cr), both in serum and organs were detected by the corresponding ELISA kits according to the standard protocols provided by the suppliers.

### Detections of Marrow Micronucleus Cell Rates and White Blood Cell Count

The marrow micronucleus cell rate (MMCR) of each group was evaluated from H&E section. For white blood cell count, 20.0 μL of anticoagulated blood from each mouse was employed to count white blood cells (WBCs).

### Immunohistochemical Analysis

Immunohistochemistry was carried out to detect the expression of Bax, Bcl-2, caspase-3, and survivin *in ex vivo* tumor tissues, which were performed as described previously (Dai et al., 2011; Dolka et al., 2016).

### Statistical Analysis

All experiments were carried out at least three times. All data are presented as mean ± standard deviation (SD) and analyzed for statistical significance using SPSS (Version 13.0, SPSS Inc., Chicago, IL, United States). ^&^*P <* 0.05 was considered statistically significant, ^#^*P <* 0.01 and ^∗^*P <* 0.001 were considered highly significant, respectively.

## Results and Discussion

### Preparation and Characterization of DOX-Loaded Micelles

The unsatisfactory stability of micelles is still an obvious challenge for their extensive application in controlled drug delivery. Herein, pairs of enantiomeric copolymers of PEG–PDLA and PEG–PLLA were employed as matrices to enhance the stability of micelles via stereocomplex interaction. In aqueous solution, PEG–PDLA, PEG–PLLA and an equimolar mixture of them could self-assemble into micelles due to their amphiphilic nature. They were noted as PDM, PLM, and SCM, respectively. As depicted in **Scheme 1**, DOX was encapsulated by PDM, PLM, or SCM by nanoprecipitation. In the previous work, all of these DOX-loaded micelles showed clear spherical morphologies and the average diameters of the micelles were about 100, 90, and 80 nm, respectively. The appropriate sizes of these micelles made them suitable candidates to selectively accumulate at tumor sites by the EPR effect.(Kobayashi et al., 2014; Liu et al., 2014) Moreover, all the loaded micelles exhibited excellent stability both in PBS and bovine serum albumin (BSA) solutions.(Wang et al., 2015a). In addition, critical micellization concentration (CMC) is one of the key parameters to describe the physical properties of micelles. The CMCs of PDM, PLM, and SCM were 0.063, 0.052, and 0.045 g/L, respectively (Liu et al., 2014). The CMC value of SCM was lower than that of PDM and PLM, induced by the stereocomplex interaction, which played an important role in the stability of the micelle.

### Intracellular DOX Release and Cell Viability Assays

**FIGURE 1:**
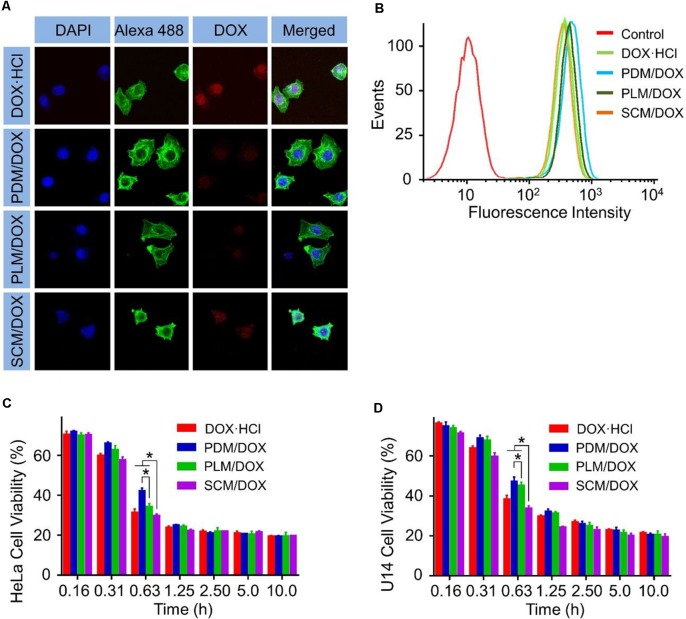
*In vitro* cellular internalization and cytotoxicity of DOX-loaded micelles on HeLa cells. **(A)** CLSM study of HeLa cells after 2 h incubation with DOX⋅HCl and DOX-loaded micelles (Scale bar = 10 μm). **(B)** FCM study of HeLa cells after 2 h incubation with DOX⋅HCl and DOX-loaded micelles. **(C)** HeLa cell viability and **(D)** U14 cell viability after treatment with DOX⋅HCl and DOX-loaded micelles for 48 h. Data are presented as mean ± SD (*n* = 8; ^∗^*P <* 0.001).

The cell uptake and intracellular release behaviors of DOX-loaded micelles were employed through CLSM and FCM. As shown in **Figure 1A**, after 2 h of incubation, the intracellular DOX fluorescences of the groups treated with DOX-loaded micelles were weaker than those cultured with free DOX⋅HCl. The different intracellular cell uptake behavior of DOX-loaded micelles and free DOX⋅HCl. was most likely due to the manner of free DOX⋅HCl enters cells was diffusion, which was faster than the endocytosis of DOX-loaded micelles (Li et al., 2014). Furthermore, the SCM/DOX group showed higher DOX fluorescences than PDM/DOX and PLM/DOX groups. This might be related to the slower extracellular DOX release and more efficient intracellular DOX release of SCM/DOX induced by stereocomplex interaction (Liu et al., 2014). These results were further confirmed by FCM analyses. In **Figure 1B**, the intracellular DOX fluorescence intensity of SCM/DOX group was higher than that in PDM/DOX and PLM/DOX group, while lower than free DOX⋅HCl. Both the CLSM and FCM verified the effective internalization of DOX-loaded micelles by HeLa cells, especially the SCM/DOX.

MTT assays in HeLa cells and U14 cells revealed that the SCM/DOX displayed significant cell killing activity after 48 h incubation (**Figures 1C,D**). It should be noted that the half maximal inhibitory concentrations (IC_50_) values for SCM/DOX against HeLa, and U14 cells were 0.47 and 0.50 μg mL^-1^, respectively, which were lower than those obtained with the PDM/DOX, PLM/DOX, and free DOX⋅HCl [(IC_50_ = 0.60, 0.52, and 0.49 μg mL^-1^, HeLa cells) and (IC_50_ = 0.65, 0.73, and 0.68 μg mL^-1^, U14 cells)]. This higher cell killing activity of SCM/DOX was attributed to the enhanced cellular uptake via stereocomplex interaction and the improved intracellular DOX release.

### *In vivo* Biodistribution of DOX-Loaded Micelles at Tumor Sites

**FIGURE 2:**
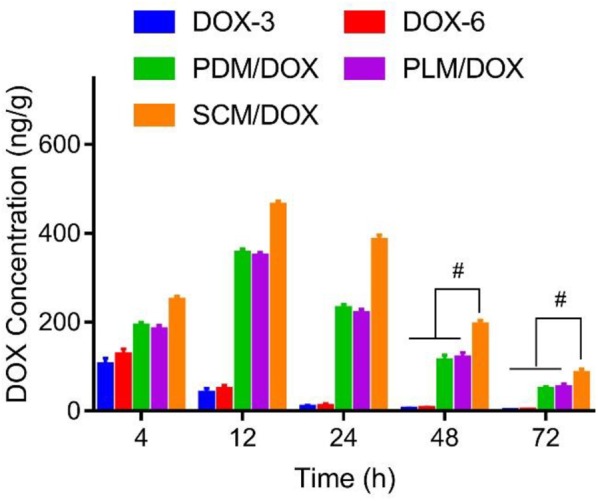
*In vivo* biodistribution of DOX⋅HCl, or DOX-loaded micelles at 4, 12, 24, 48, and 72 h after injection, respectively. Data were presented as mean ± SD (*n* = 6), ^#^*P <* 0.01.

The *in vivo* DOX biodistribution at tumor sites was explored by treating tumor-bearing mice with free DOX⋅HCl and DOX-loaded micelles, respectively. As shown in **Figure 2**, free DOX⋅HCl was rapidly distributed in tumors at 4 h and rapidly eliminated at 12 h. The signal of DOX in tumors was negligible after 24 h. In contrast, DOX in the DOX-loaded micelles groups showed a much slower elimination. The concentrations of DOX in the primary tumors gradually reached to the maximum contents at 12 h post injection and was significantly increased compared to the free DOX⋅HCl groups (both the D0X-3 and DOX-6 groups). Until 72 h later, the DOX-loaded micelles groups still showed high DOX concentrations in tumor than free DOX⋅HCl groups (*P <* 0.01). Furthermore, compared to PDM/DOX and PLM/DOX, SCM/DOX exhibited the highest DOX concentration in tumor sites, which further verified the enhanced cellular uptake via stereocomplex interaction. This long-lasting delivery of DOX-loaded micelles was beneficial to the treatment of the tumor. These results could be due to the improved EPR effect and the decreased reticulo-endothelia system (RES) elimination by the PEG coating.

### *In vivo* Antitumor Efficacy Measurement

**FIGURE 3:**
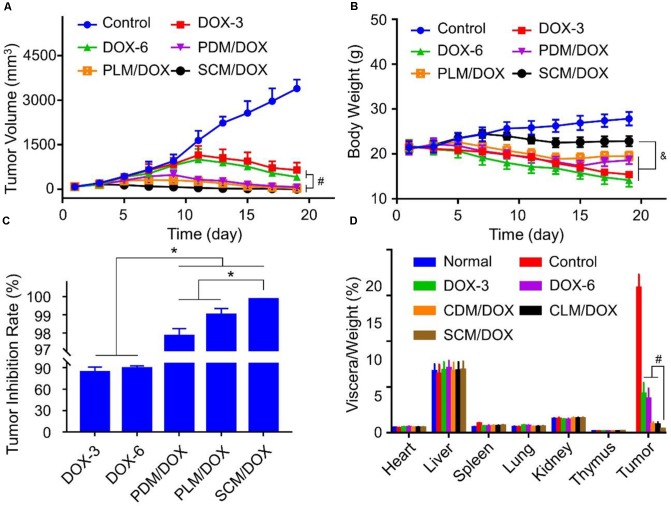
*In vivo* antitumor effect of DOX-loaded micelles in the U14 tumor-bearing BALB/c mice. **(A)** Tumor growth profles, **(B)** body weight changes, **(C)** quantitative analysis of the tumor inhibition rates, and **(D)** organ indices of U14 cervical cancer-allografted mice after injected with NS, DOX⋅HCl, or DOX-loaded micelles. Each set of data is represented as mean ± SD (*n* = 8; ^&^*P <* 0.05, ^#^*P <* 0.01, ^∗^*P <* 0.001).

**FIGURE 4:**
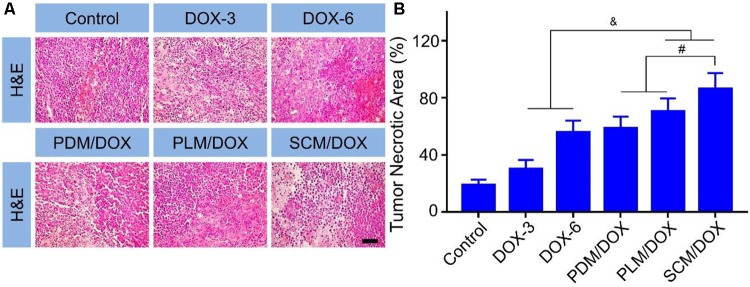
Histopathological analysis of tumor tissues. **(A)** H&E staining (Scale bar = 50 μm) of the tumor tissues from the mice. **(B)** Quantitative analysis of the tumor necrotic area of each group. Each set of data is represented as mean ± SD (*n* = 3; ^&^*P <* 0.05, ^#^*P <* 0.01).

The *in vivo* antitumor efficacy of DOX-loaded micelles was evaluated using a U14 subcutaneous cervical carcinoma model in BALB/c mice. As shown in **Figure 3A**, in comparison to free DOX⋅HCl, DOX-loaded micelles showed a stronger tumor inhibition effect, probably due to the enhanced accumulation at tumor site through EPR effect.(Wang et al., 2015a,b) Importantly, SCM/DOX group exhibited better antitumor efficacy than PDM/DOX and PLM/DOX, which might be related to the improved stability of SCM/DOX, suggesting less extracellular DOX release and greater internalized in the tumor cells. Moreover, the tumor inhibition rates of PDM/DOX, PLM/DOX, and SCM/DOX were 97.8 ± 0.40, 99.0 ± 0.35, and 99.8% ± 0.02%, which were higher than that of DOX-3 and DOX-6 groups (*i.e.*, 83.7 ± 7.01 and 89.2 ± 3.21%; *P <* 0.001) (**Figure 3C**). Although free DOX⋅HCl also showed some tumor inhibition effect, severe body weight loss was observed during therapy (**Figure 3B**), indicating its serious systemic toxicity to mice. In contrast, the DOX-loaded micelles-treated groups did not exhibit significant body weight loss, indicating satisfactory tolerance of mice to DOX-loaded micelles. In addition, organ indices were calculated by the weight ratios between organs (mg) and the whole body (g) to provide a general impression of toxicity. As shown in **Figure 3D**, no obvious difference was observed in the heart, liver, spleen, lung, kidney, and thymus indices among all the groups, indicating the DOX-loaded micelles would not lead to severe systemic toxicities when they were used *in vivo*. Conversely, the tumor indices of the DOX-loaded micelles groups, especially the SCM/DOX, were much lower than that of free DOX⋅HCl groups. These indices were consistent with the tumor inhibition rates. H&E staining of tumor sections was performed to investigate the fate of tumor cells after experiencing treatment in the four groups (**Figure 4A**). As shown, tumor tissues from mice in control group showed no obvious necrosis or apoptosis and the tumor cells retained their normal morphology and nuclear structure, indicating that the tumor cells in control group proliferated quickly. In contrast, tumor tissues showed various degrees of necrosis in the free DOX⋅HCl and DOX-loaded micelles-treated groups. Furthermore, DOX-loaded micelles-treated groups showed larger necrosis area than free DOX⋅HCl-treated groups. In detail, the quantitative necrosis area of PDM/DOX, PLM/DOX, and SCM/DOX-treated groups were 58.6, 70.3, and 86.2% (**Figure 4B**), respectively, which were higher than that in DOX-3-treated group (30.1%) and DOX-6-treated group (55.7%) (*P <* 0.05). This observation indicated the effective antitumor efficacy of DOX-loaded micelles, especially the SCM/DOX.

**FIGURE 5:**
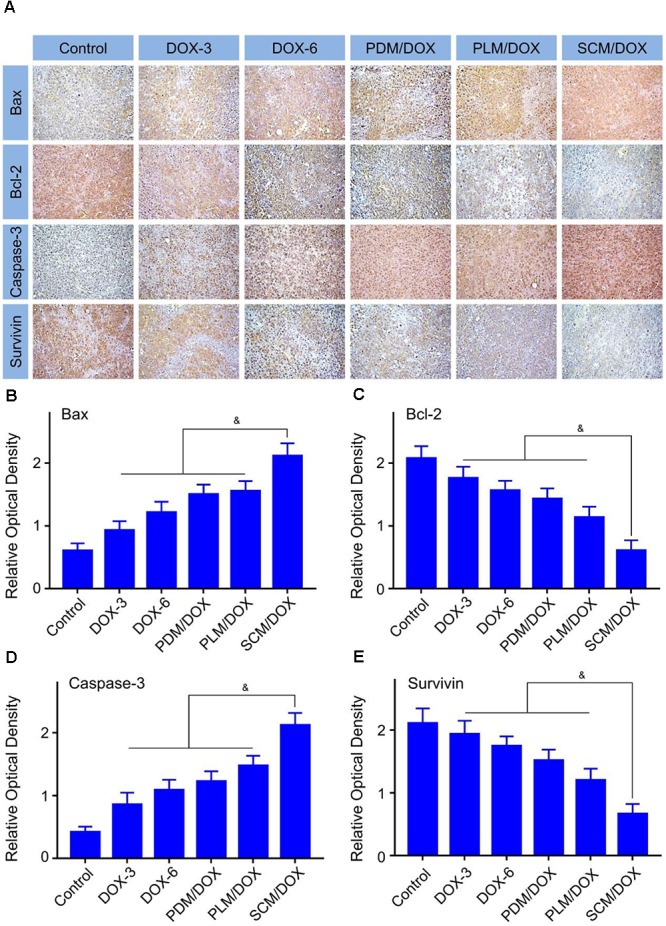
Immunohistochemical analysis of tumor tissues. **(A)** Immunohistochemical (*i.e.*, Bax, caspase-3, survivin, and Bcl-2) analyses (Scale bar = 50 μm) of tumor tissues sections. Relative positive area of tumor sections from Bax **(B)**, Bcl-2 **(C)**, caspase-3 **(D)**, and survivin **(E)** after all treatments. Each set of data is represented as mean ± SD (*n* = 3; ^&^*P <* 0.05).

Immunohistochemical staining analysis was performed to further verify the antitumor efficacy of DOX-loaded micelles. As shown in **Figure 5A**, the pro-apoptotic protein Bax (brown) and caspase-3 (brown) signals of from the tumor cells that received the treatment of DOX-loaded micelles, especially the SCM/DOX, were much higher than free DOX⋅HCl groups. In detail, the SCM/DOX-treated group showed 2.3, 1.7, 1.4, and 1.4 times Bax signal (**Figure 5B**) (*P <* 0.05) and 2.5, 2.0, 1.7, and 1.4 times caspase-3 signal than DOX-3, DOX-6, PDM/DOX, and PLM/DOX (**Figure 5D**) (*P <* 0.05), respectively. In contrast, the expression of antiapoptotic protein Bcl-2 (brown) decreased significantly in the DOX-loaded micelles-treated groups, especially the SCM/DOX group. The Bcl-2 signal of SCM/DOX group was 0.7, 0.6, 0.6, and 0.5-fold decrease compared with DOX-3, DOX-6, PDM/DOX, and PLM/DOX (*P <* 0.05), respectively (**Figure 5C**). In addition, survivin, which can improve the survival of tumor cells primarily, was also used to evaluate cell survival. As shown in **Figure 5E**, the expressions of survivin (brown) decreased in the DOX-loaded micelles-treated groups, especially the SCM/DOX group. The SCM/DOX group showed a 0.7, 0.6, 0.6, and 0.4-fold decrease of survival signal compared with DOX-3, DOX-6, PDM/DOX, and PLM/DOX (*P <* 0.05), respectively. These data clearly demonstrated that our DOX-loaded micelles, especially SCM/DOX, could serve as highly effective nano therapeutic agents.

### *In vivo* Security Evaluation

**FIGURE 6:**
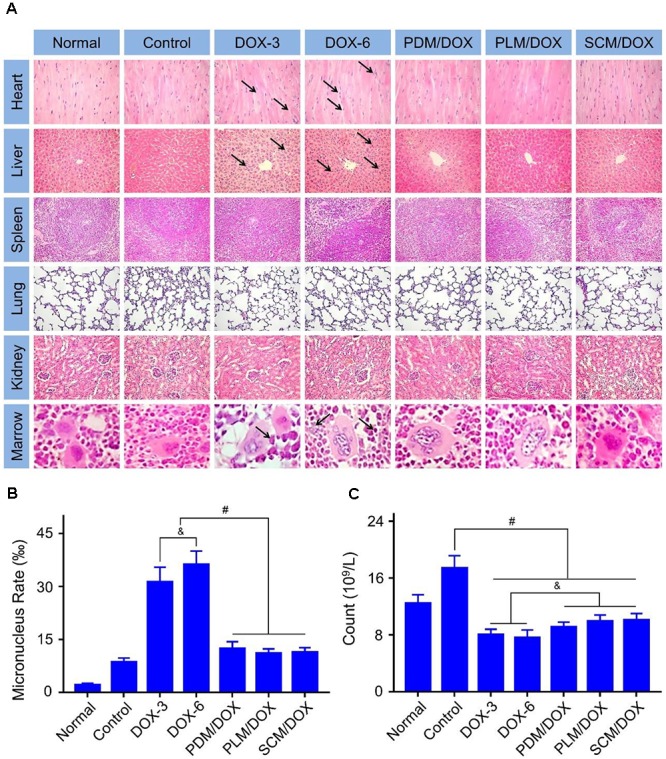
Histopathological examination of visceral tissues. **(A)** Histopathological analyses (Scale bar = 50 μm) of internal tissues from normal mouse, or U14 cervical cancer-allografted BALB/c mice after treatment with NS, DOX⋅HCl, or DOX-loaded micelles. Black arrows indicated the occurrence of pathological changes. **(B)** Micronucleus rate and **(C)** WBC count of normal mouse or U14 cervical cancer-allografted BALB/c mice after all treatments. Each set of data is represented as mean ± SD (*n* = 3; ^&^*P <* 0.05, ^#^*P <* 0.01).

The *in vivo* toxicity of DOX was detected by the histopathological analysis. As shown in **Figure 6A**, the obvious accumulation of neutrophils and myocardial fiber breakage were detected in the heart of free DOX⋅HC-treated groups, especially the DOX-6 group, indicating the evident cardiotoxicity of free DOX⋅HCl. All of these damages were pointed out by black arrows in **Figure 6A**. In contrast, DOX-loaded micelles-treated groups did not show neutrophils accumulation and the myocardial cells lined in order and their sarcolemma-maintained integrity, which could be due to the decreased distribution of DOX in heart. Besides, free DOX⋅HCl-treated group showed hepatotoxicity, which was revealed in the microregional necrosis of hepatocytes. In contrast, less structural disturbance was showed in the DOX-loaded micelles-treated groups. In addition, free DOX⋅HCl-treated group also showed nephrotoxicity, as judged through the shriveled glomerular and unclear cell morphology. On the contrary, the structure of the kidney in the DOX-loaded micelles-treated groups was intact. All the data demonstrated the decreased systematic toxicity of DOX-loaded micelles, which could be due to the decreased DOX release from the micelles during blood circulation and less DOX accumulated at normal tissues and organs. No obvious pathological change was found in the spleens and lungs of the DOX-loaded micelles groups, indicating the good biocompatibility of them.

The damages of small molecular chemotherapeutic drugs to chromosomes can be reflected in the increase of MMCR.(Chen et al., 1994) As shown in **Figure 6A**, various quantities of bone marrow mononuclear cells were observed in different groups. The specific quantitative proportions of them showed that the MMCRs in DOX-3 and DOX-6 groups were ascendant, while the MMCRs of DOX-loaded micelles-treated groups were slightly ascendant (*P <* 0.01) (**Figure 6B**). The results were in accordance with the histopathological analysis of tissues and organs, which further demonstrated the detoxification of DOX-loaded micelles.

It is reported that the count of WBC can reflect the influence of chemotherapy on the immune status.(Homma et al., 2014) In **Figure 6C**, the WBC count of the control group significantly raised in comparison to other groups, indicating that the treatments with free DOX⋅HCl and DOX-loaded micelles could effectively eliminate the inflammation caused by tumor (*P <* 0.01). In addition, the DOX-loaded micelles showed a more efficient anti-inflammatory efficacy than free DOX⋅HCl (*P <* 0.05). All the data further verified the advantages of DOX-loaded micelles in the application of anti-cervical carcinoma than free DOX⋅HCl.

**FIGURE 7:**
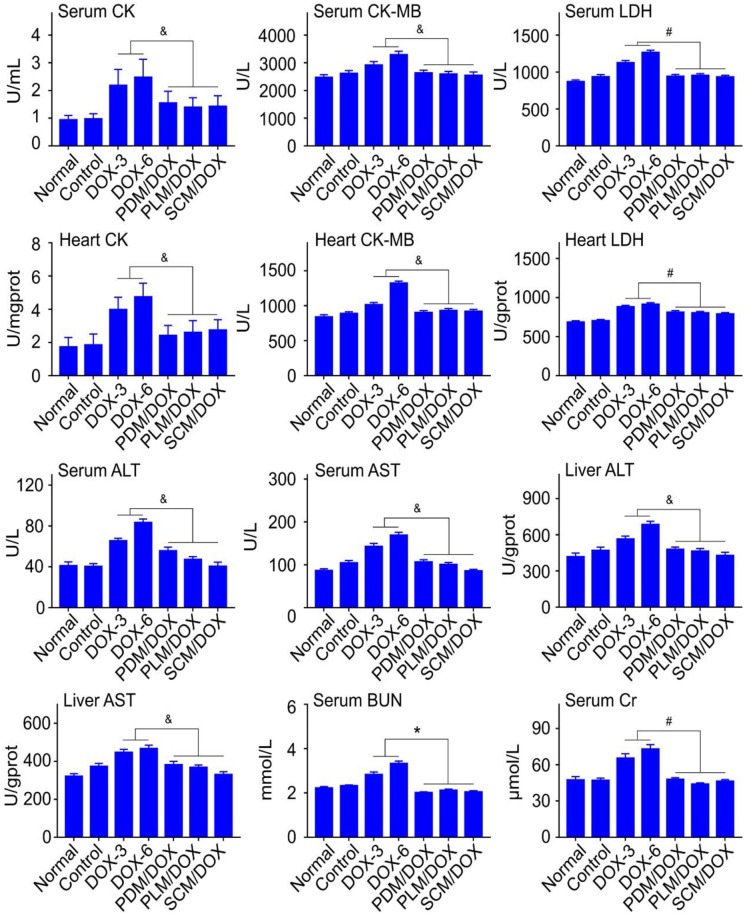
Biochemical parameter assays for safety evaluation. Examinations of CK, CK-MB, LDH, ALT, AST, BUN, and Cr in serum and corresponding internal organs of normal mice or U14 cervical cancer-allografted BALB/c mice after treatment with NS, DOX⋅HCl, or DOX-loaded micelles. Each set of data is represented as mean ± SD (*n* = 3; ^&^*P <* 0.05, ^#^*P <* 0.01, ^∗^*P <* 0.001).

Clinical chemical parameters, including CK, CK-MB, LDH, ALT, AST, BUN, and Cr were tested to further demonstrate the security of DOX-loaded micelles *in vivo*. As shown in **Figure 7**, the relevant parameters for both serum and organs were obviously raised in free DOX⋅HCl-treated groups, especially the DOX-6 group, which indicated that free DOX⋅HCl caused obvious damage to heart, liver, and kidney (*P <* 0.05). In contrast, all the DOX-loaded micelles-treated groups, especially the SCM/DOX group, showed negligible changes of relevant parameters, which were almost the same with normal group. The data verified that all the DOX-loaded micelles, especially the SCM/DOX could minimize the damage of DOX to the body. All of the up-mentioned results were consistent with the body weight changes and immunohistochemistry results of tissues and organs. All these results demonstrated that all of the DOX-loaded micelles, especially the SCM/DOX, were relatively safe and could be potentially applied in clinical studies in the future.

## Conclusion

In this study, DOX-loaded polylactide based micelles (PDM/DOX and PLM/DOX) and stereocomplex micelle (SCM/DOX) were fabricated. *In vitro* studies showed that SCM/DOX increased the cellular uptake compared to PDM/DOX and PLM/DOX and exhibited the strongest cytotoxicity against HeLa cells, which was even stronger than free DOX⋅HCl. Furthermore, in a mouse U14 cervical carcinoma model, SCM/DOX also exhibited the most efficient antitumor efficacy compared to either PDM/DOX, PLM/DOX or free DOX⋅HCl. Importantly, all the DOX-loaded micelles, especially the SCM/DOX could obviously alleviate the systemic toxicity of DOX. Therefore, the stereocomplex micelle could serve as a promising nanodrug delivery system with high systemic safety for the future cervical carcinoma therapy.

## Author Contributions

These studies were conceived of and designed by all authors. Experiments were performed by KN and YY. Data analysis, data interpretation, manuscript preparations were done by MX, CG, YG, and JW.

## Conflict of Interest Statement

The authors declare that the research was conducted in the absence of any commercial or financial relationships that could be construed as a potential conflict of interest.
